# The therapeutic effect and mechanism of melatonin on osteoarthritis: From the perspective of non-coding RNAs

**DOI:** 10.3389/fgene.2022.968919

**Published:** 2022-10-04

**Authors:** Shuai Li, Haibo Si, Jiawen Xu, Yuan Liu, Bin Shen

**Affiliations:** Department of Orthopedics, Orthopedic Research Institute, West China Hospital, Sichuan University, Chengdu, Sichuan, China

**Keywords:** osteoarthritis, melatonin, microRNA, circular RNA, long non-coding RNA, circadian clocks, epigenetics

## Abstract

Osteoarthritis (OA) is a slowly progressing and irreversible joint disease. The existing non-surgical treatment can only delay its progress, making the early treatment of OA a research hotspot in recent years. Melatonin, a neurohormone mainly secreted by the pineal gland, has a variety of regulatory functions in different organs, and numerous studies have confirmed its therapeutic effect on OA. Non-coding RNAs (ncRNAs) constitute the majority of the human transcribed genome. Various ncRNAs show significant differentially expressed between healthy people and OA patients. ncRNAs play diverse roles in many cellular processes and have been implicated in many pathological conditions, especially OA. Interestingly, the latest research found a close interaction between ncRNAs and melatonin in regulating the pathogenesis of OA. This review discusses the current understanding of the melatonin-mediated modulation of ncRNAs in the early stage of OA. We also delineate the potential link between rhythm genes and ncRNAs in chondrocytes. This review will serve as a solid foundation to formulate ideas for future mechanistic studies on the therapeutic potential of melatonin and ncRNAs in OA and better explore the emerging functions of the ncRNAs.

## Introduction

Osteoarthritis (OA), a slowly progressing disease with irreversible structural changes, can develop and show the clinical manifestation of chronic pain. Active early treatment can delay the progress of the OA (Hunter and Bierma-Zeinstra, 2019). OA can not only cause local joint symptoms, reducing the quality of life of patients but also coexists with heart disease, diabetes, and mental health problems, which will significantly increase the incidence of adverse events such as hip fractures, bringing a considerable burden to patients, families, and society (Wang et al., 2021a). OA is the leading cause of disability in the elderly (Hunter and Bierma-Zeinstra, 2019). More than 500 million people worldwide suffer from OA, accounting for 7% of the global population (Hunter et al., 2020). This number may be exacerbated by the aging population and the growing trend of obesity (Hunter et al., 2020). Before progressing to the terminal stage of OA that can only be effectively solved by joint replacement, the mainstream drug treatment of OA is to inhibit inflammation and reduce pain. New treatment methods are constantly being explored, but significant progress has not been made compared with the treatment of many other musculoskeletal and chronic non-communicable diseases.

Melatonin (N-acetyl-5-methoxytryptamine) is an endogenous indole hormone mainly secreted by the mammalian pineal gland. Its production and secretion duration directly depends on the length of the night. In other words, its secretion has a circadian rhythm (Cipolla-Neto and Amaral, 2018). With the aging of the body, the secretion of melatonin also gradually decreases (Hardeland, 2012). Coincidentally, the incidence rate of OA is also increasing (Neogi and Zhang, 2013; Quicke et al., 2022). Melatonin can regulate a variety of rhythm genes. Previous studies mainly focused on melatonin regulation in the nervous system, such as regulating circadian rhythm and promoting sleep. However, in recent years, the functions of melatonin have been gradually explored, such as participating in anti-tumor, anti-oxidation, regulating circadian rhythm, regulating immunity, regulating inflammatory response, promoting wound healing, and tissue regeneration (Hardeland, 2019). The disorder of circadian rhythm will hinder the normal production of melatonin, leading to a high level of inflammatory factors in body fluid, and making the body stay in a state of chronic inflammation, suggesting the relationship between melatonin and chronic inflammatory diseases (Zhang et al., 2019b).

Non-coding RNAs (ncRNAs) can’t encode proteins, but they have properties that affect normal gene expression and disease progression, making them new targets for exploring how drugs work (Matsui and Corey, 2017). As a research hotspot in recent years, numerous ncRNAs and their role in OA have been found with the development of gene sequencing technology. MicroRNA (miRNAs), circular RNAs (circRNAs), and long non-coding RNAs (lncRNAs) are ncRNAs that play a major regulatory role in OA (Wu et al., 2019). Numerous studies have also proved the therapeutic strategy of introducing exogenous ncRNAs into the joint to regulate OA in the past few years (Liang et al., 2020). Meanwhile, innumerable substances with therapeutic effects on OA, including melatonin, have achieved their therapeutic effect by regulating ncRNAs (Qiu et al., 2020).

## The role of melatonin in the skeletal muscle system

The regulatory effect of melatonin on bone and cartilage has been found in the musculoskeletal system, especially in degenerative diseases. A previous study found that In patients with intervertebral disc degeneration (IVDD), melatonin can promote proliferation, induce autophagy and inhibit apoptosis of annulus fibrosus cells (Hai et al., 2019). Moreover, melatonin regulates the expression of autophagy-related proteins by inhibiting the function of miR-106a-5p (Hai et al., 2019). In addition, melatonin can also affect bone metabolism by regulating the function of osteoblasts and osteoclasts, and its role in osteoporosis (Zhang et al., 2016; Han et al., 2021), fracture healing (Histing et al., 2012), and other diseases have also been confirmed.

Melatonin has been found to protect chondrocytes by inhibiting inflammation, promoting matrix synthesis, and promoting cartilage differentiation of bone marrow mesenchymal stem cells (BMSCs) (Wu et al., 2018) and other ways (Jorge et al., 2021). The previous study (Han et al., 2021a) found that melatonin plays a protective role on synovial cells by alleviating the effect of D-galactose (D-gal) on synovial cells by upregulating silent information regulator 1 (SIRT1), finally promoting hyaluronic acid synthesis and interrupting cell aging. The imbalance of endogenous hormones, including melatonin, leads to the expression of inflammation-related cytokines and matrix degradation-related proteases in articular cartilage, causing cartilage erosion, synovitis, and osteophyte formation (Hossain et al., 2019). Compared with other drugs, melatonin has a wide range of advantages and almost no side effects (Lu et al., 2021b).

## The interaction of melatonin with ncRNAS

The relationship between melatonin and ncRNAs has been verified in inflammation, oxidative stress, cancer, aging, energy consumption, obesity, type 2 diabetes, neuropsychiatric disorders, and neurogenesis (Hardeland, 2014; Mori et al., 2016). When melatonin regulates its target genes, the level of specific ncRNAs also changes accordingly, suggesting that melatonin can exert its potential by regulating ncRNAs. A previous study (Han et al., 2020) found that melatonin induced the increase of miR-149 and inhibited the expression of pro-inflammatory cytokine tumor necrosis factor-α (TNF-α) and extracellular matrix (ECM) components such as type I collagen and fibronectin in the mouse model of hindlimb ischemia, to produce anti-inflammatory and anti-fibrosis protective effect on ischemic tissue. Meanwhile, this protective effect can be blocked by inhibiting miR-149. Che et al. (2020) suggested that melatonin could regulate the lncRNAs-miRNAs axis (Che et al., 2020). That is, melatonin administration significantly ameliorated cardiac dysfunction and reduced collagen production by inhibiting transforming growth factor-β (TGF-β1) /SMAD signaling and nucleotide-binding domain and leucine-rich repeat containing PYD-3 (NLRP3) inflammasome activation in the diabetes mice model (Che et al., 2020).

The researchers found that some miRNAs were highly enriched in the pineal gland, and a few miRNAs were enriched differently between day and night which is similar to the rhythm of pineal gland secretion of melatonin (Clokie et al., 2012). Some specific ncRNAs can further regulate the circadian rhythm by regulating the expression of enzymes related to the synthesis and secretion of melatonin. A previous study found that miR-483 directly targets the mRNA of arylalkylamine N-acetyltransferase (AANAT), the main rate-limiting enzyme for melatonin formation, and inhibited melatonin synthesis (Clokie et al., 2012). Similarly, Zheng et al. (2022) found that circRNA-WNK2 was highly expressed at night and could competitively bind to miR-328a-3p and reduce the expression of miR-328a-3p, thereby enhancing the expression of AANAT inhibited by miR-328a-3p, and ultimately stimulated melatonin secretion (Zheng et al., 2022). Meanwhile, the expression of ncRNAs in the pineal gland is regulated by signals from the suprachiasmatic nucleus. Some ncRNAs can also regulate the expression of related receptors on melatonin target cells. As in a genetic mouse model of atherosclerosis, a high-fat diet induces miR-29 expression and targets and inhibits MT1’s expression by binding to MT1 mRNA 30-UTR of melatonin, showing the protective effect of reducing the molecular and cellular damage function of cardiac ischemia-reperfusion (Zhu et al., 2014). Other ncRNAs, such as piR015520, can also regulate the expression of melatonin-related genes (Esposito et al., 2011). Zheng et al. (2021) constructed a competing endogenous RNAs (ceRNAs) network composed of circRNAs, miRNAs, and mRNAs by analyzing and screening circRNAs and miRNAs differentially expressed in the pineal gland of rats, which may play an essential regulatory role in the circadian rhythm and melatonin secretion of the pineal gland (Zheng et al., 2021).

## The role of melatonin in osteoarthritis

**FIGURE 1:**
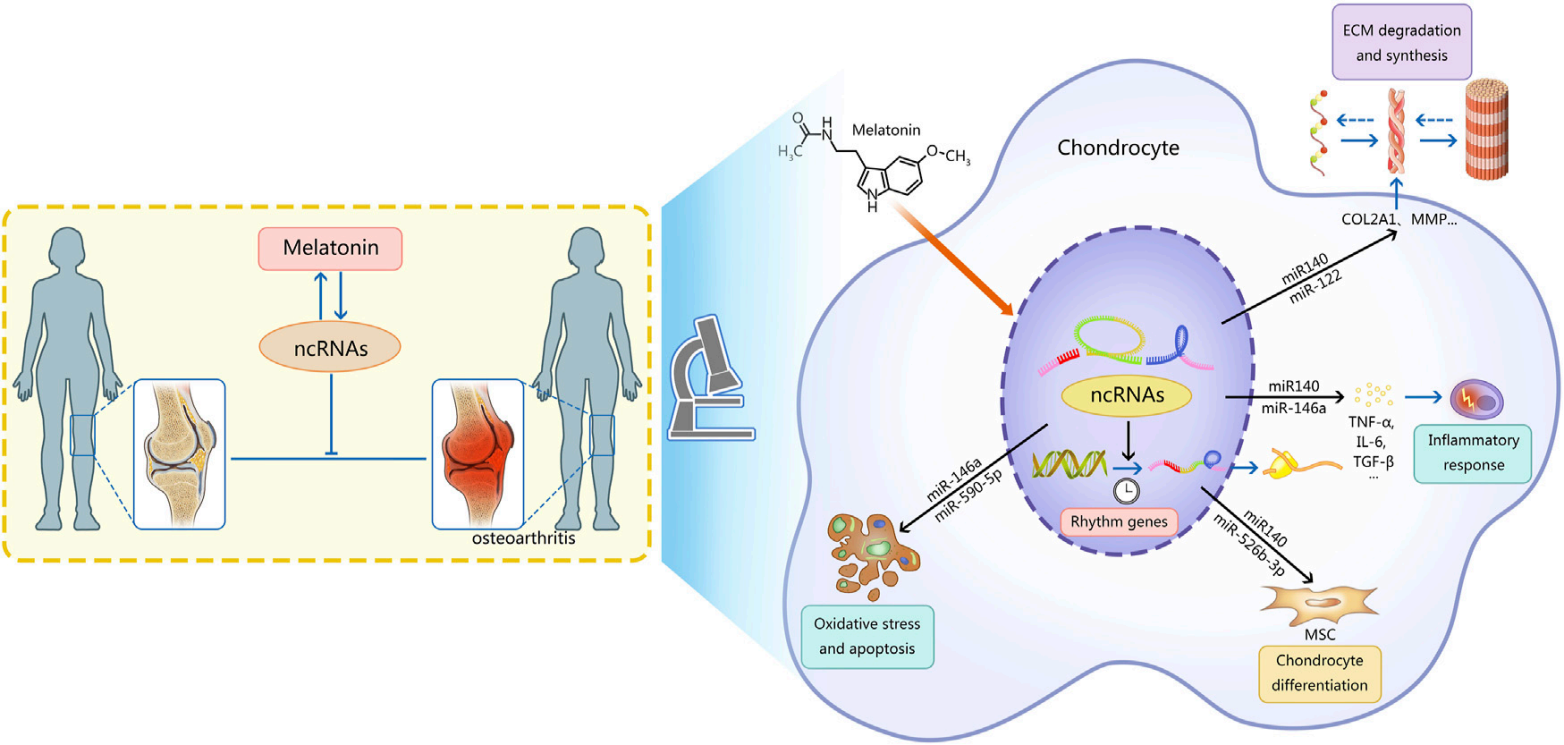
This figure describes the mechanism of interaction between ncRNAs and melatonin in osteoarthritis. Note: Melatonin can regulate OA by regulating the level of ncRNAs, which is mainly reflected in five aspects: 1) regulating the degradation and synthesis of the chondrocyte matrix; 2) regulating the oxidative stress and apoptosis of chondrocytes; 3) regulating the differentiation of chondrocytes; 4) regulating rhythm genes; 5) regulating inflammatory mediators.Ultimately the progression of osteoarthritis is delayed. Meanwhile, ncRNAs can regulate the synthesis and function of melatonin. AANAT, arylalkylamine N-acetyltransferase; ncRNAs, non-coding RNAs; MSC, mesenchymal stem cells; MMP, matrix metalloproteinase; ADAMTS, a disintegrin metalloproteinase with thrombospondin motifs; ECM, extracellular matrix; TNF-α, tumor necrosis factor-α; IL-6, interleukin 6; TGF-β, transforming growth factor-β.

Recently, the therapeutic effects of melatonin and ncRNAs in OA have been gradually discovered. Exogenous melatonin can stimulate the growth of chondrocytes and regulate the expression of cartilage-related genes. At the same time, chondrocytes can synthesize a small amount of melatonin (Fu et al., 2019). Endogenous and exogenous melatonin can regulate cartilage growth and maturation through MT1 and MT2 receptors (Fu et al., 2019). Melatonin can regulate OA by adjusting the level of ncRNAs, which is mainly reflected in five aspects (Figure 1): 1) regulating the degradation and synthesis of the chondrocyte matrix; 2) regulating the oxidative stress and apoptosis of chondrocytes; 3) regulating the differentiation of chondrocytes; 4) regulating rhythm genes; 5) regulating inflammatory mediators. In addition, melatonin can also alleviate the adverse effects of other drugs on OA.

### Regulation of extracellular matrix degradation and synthesis

It is widely believed that the dysregulation of cartilage matrix synthesis and degradation is the cause of OA (Guo et al., 2017). The main components of the cartilage matrix are collagen and proteoglycans. Collagen type II is the most abundant component in the cartilage matrix (Xie et al., 2021). The latest research proposes the concept of the pericellular matrix (PCM) and regards the changes that occur in PCM as initiating or progressive factors for OA (Guilak et al., 2018). *In vitro* experiments, melatonin was found to increase the expression of chondrogenesis marker genes such as COL2A1, SOX9, and aggrecan, which are major components of the ECM of cartilage (Pei et al., 2009).

The enzymes related to cartilage ECM are enhanced during OA, mainly including matrix metalloproteinase (MMP) that degrades type II collagen and a disintegrin metalloproteinase with thrombospondin motifs (ADAMTS). ADAMTS is a family of 19 secreted metalloproteinases involved in various developmental and homeostatic processes (Kelwick et al., 2015). And several members of the ADAMTS family have been shown to degrade the cartilage proteoglycans (Verma and Dalal, 2011). Melatonin is a multifaceted regulator of MMP gene expression and activity (Swarnakar et al., 2011). In addition, melatonin can inhibit ADAMTS activity by regulating miRNAs and has an inhibitory effect on cartilage ECM degradation (Zhang et al., 2019c).

### Regulation of oxidative stress and apoptosis

Reactive oxygen species (ROS) is the leading cause of chondrocyte damage and OA development (Blanco et al., 2011). Oxidative stress caused by ROS can oxidize and subsequently disrupt cartilage homeostasis, promoting catabolism by inducing cell death and damaging many components of the joint (Lu et al., 2021a). In addition, oxidative stress also destroys the cartilage matrix by inhibiting ECM synthesis and upregulating potential ECM-degrading related enzymes (Lu et al., 2021a). Mitochondria are the primary source of intracellular reactive oxygen species, and mitochondrial dysfunction plays a vital role in chondrocyte autophagy (Hosseinzadeh et al., 2016). Melatonin and its derivatives are a broad range of free radical scavengers and antioxidants due to their potential to scavenge ROS and reactive nitrogen species (RNS) and promote glutathione and antioxidant enzymes expression and activity (Reiter et al., 2010). In addition, melatonin can regulate chondrocyte apoptosis by regulating TNF-α, interleukin 6 (IL-6), SIRT, TGF-β, and other signaling pathways (Reiter et al., 2010). Exosomes secreted by adipose tissue-derived stem cells (ADSCs) can significantly reduce H_2_O_2_-induced apoptosis of articular chondrocytes and promote chondrogenesis by upregulating miR-145 and miR-221 (Zhao et al., 2020). Melatonin indirectly regulates the downstream signal miR-210 by scavenging reactive oxygen species, suggesting the role of melatonin and miRNAs in oxidative stress-related diseases (He et al., 2020). Melatonin was found to inhibit H_2_O_2_-induced oxidative stress and exert a protective effect on chondrocytes by maintaining mitochondrial redox homeostasis and regulating autophagy (Chen et al., 2021c). Changes in the expression of miR-223 affect the metabolic status of cells and alter the conditions of apoptosis and proliferation of cells, whereas melatonin reduces the expression of miR-223 in wild-type (WT) aged mice (Sayed et al., 2021).

### Regulation of chondrocyte differentiation

As a pleiotropic regulator, melatonin has been shown to regulate many biological behaviors of stem cells in numerous studies. BMSCs have inherent chondrogenic differentiation potential and are an ideal choice for cartilage regeneration. Previous studies speculated that BMP/SMAD signaling pathway is involved in the melatonin-induced chondrogenic differentiation of human mesenchymal stem cells (hMSCs) (Zheng et al., 2015). The treatment of early OA often happens in an inflammatory microenvironment, and it may be more practical to promote the chondrogenic differentiation of BMSCs in an inflammatory environment. Melatonin was found to alleviate chondrogenesis of human bone marrow mesenchymal stem cells (hBMSCs) inhibited by IL-1β treatment by restoring chondrocyte size and cartilage matrix accumulation, maintaining metabolic balance, and reducing apoptosis (Gao et al., 2018). Further studies revealed that melatonin rescued chondrogenic differentiation inhibited by IL-1β mainly through inhibiting the activation of the NF-κB (nuclear factor kappa-B) signaling pathway, one of the most critical pathways involved in inflammation and apoptosis.

### Regulation of rhythm genes

The regulatory effect of the central nervous system on OA has been discovered in recent years (Morris et al., 2019). Clinical studies have shown that the clinical manifestations of OA patients, such as pain or stiffness, are rhythmic changes (Bellamy et al., 2002). As mentioned above, chondrocytes can be regulated by both exogenous melatonin and endogenous melatonin produced by themselves. Chondrocytes may synchronize the rhythm gene expression of chondrocytes with the central biological clock by producing melatonin and upregulating the expression of melatonin receptors (Fu et al., 2019). Chondrocyte rhythm genes are important regulatory targets of melatonin, and the normal expression of rhythm genes is necessary for cartilage homeostasis. The local inflammatory environment may disrupt the normal rhythm of chondrocytes by affecting the expression of rhythm genes (Haas and Straub, 2012). Knockout of specific essential rhythm genes worsens experimental OA in rats (Hashiramoto et al., 2010). For example, the expression of the rhythm gene *bmal1* was found to be decreased in the cartilage of OA patients and the cartilage of aged mice. In addition, the inhibition of targeting *bmal1* in mouse chondrocytes can make the circadian rhythm disappear and further downregulate the expression of matrix-related genes Sox 9, ACAN, and Col2a1, as well as upregulation of phosphorylated SMAD 2/3 (p-SMAD 2/3) levels, leading to progressive degeneration of articular cartilage (Dudek et al., 2016).

Rhythm genes are thought to be the bridge between melatonin and OA. The disturbances in circadian rhythms are closely related to inflammatory joint diseases. Appropriate melatonin concentrations can correct abnormal chondrocyte phenotype by restoring abnormal rhythm gene expression in OA (Reiter et al., 2010). Previous studies have found that melatonin regulates rhythmic genes through a post-translational mechanism. Melatonin may inhibit the destruction of rhythm gene transcription factors by directly inhibiting the proteasome or limiting the destruction of rhythm proteins involved in the negative feedback of gene transcription (Vriend and Reiter, 2015). Melatonin is involved in rhythm gene expression in chondrocytes in at least two ways: 1) direct inhibition of pro-inflammatory cytokine release, indirect regulation of the expression of *per1, per2, and cry2* (Jahanban-Esfahlan et al., 2018); For example, IL-1β severely disrupted circadian gene expression rhythm in cartilage (Guo et al., 2015) , and melatonin may inhibit inflammatory mediators including IL-1β and further regulate the expression of commandment genes regulated by inflammatory mediators. 2) blockade of NF-κB signalings, such as *clock* and *bmal1* (Jahanban-Esfahlan et al., 2018). Abnormal changes in cartilage specimens accompany the low-level expression of rhythm genes. In contrast, a specific dose of melatonin restored the expression of rhythm-controlling genes and corrected the abnormal chondrocyte phenotype. However, long-term use of melatonin promoted the cleavage of Receptor Activator of Nuclear Factor-κ B Ligand (RANKL) protein in the synovium, resulting in severe subchondral bone erosion, and these changes were by regulating rhythm genes (Hong et al., 2017). There are also interactions between rhythm genes and ncRNAs. Nuclear receptor subfamily 1 group D member 1 (NR1D1/Rev-erbα), a protein encoded by rhythm genes, is present in adipocytes, macrophages, and muscle cells, controlling circadian rhythms and lipid and glucose metabolism. MiR-882 negatively regulates Rev-erbα expression by binding to Rev-erbα 3′-UTR to inhibit the translation of Rev-erbα, while melatonin may indirectly regulate Rev-erbα by reducing the expression of miR-882 (Tian et al., 2021). Na et al. (2009) predicted the relationship between miRNAs and rhythm genes. miR-181d and miR-191 could target and inhibit the expression of *clock* and *bmal1* genes, respectively, thereby regulating biological rhythms (Na et al., 2009).

### Regulation of inflammatory mediators and inflammatory responses

The regulatory effect of melatonin on inflammation is related to the state of cells (Vriend and Reiter, 2015). The adverse effects of inflammatory mediators on chondrocytes are multifaceted. The inflammatory response induced by local inflammatory mediators in the joints can lead to ROS production, inducing apoptosis, promoting collagen degradation, and inhibiting chondrocyte differentiation, finally affecting the self-repair of chondrocytes. Numerous studies have proved the anti-inflammatory effect of melatonin. The anti-inflammatory effect of melatonin is partly achieved through ncRNAs in chondrocytes (Liu et al., 2022) Previous study found that melatonin treatment enhanced mesenchymal stem cells (MSC) viability and increased the levels of anti-inflammatory miRNAs (e.g., miR-34a, miR-124, and miR-135b) in exosomes (Liu et al., 2014; Heo et al., 2020). Melatonin was found to reverse inflammatory mediator-induced inhibition of chondrogenic differentiation and modulate ROS and MMP levels. Melatonin significantly reduces the expression of miR-21, miR-146a, and miR-223, which are positively correlated with pro-inflammatory and pro-apoptotic molecules in WT mice (Sayed et al., 2021). Multiple studies have demonstrated that melatonin and ncRNAs modulate inflammatory responses in OA chondrocytes by regulating TNF-α/IL-6 levels.

### Prevent moderate adverse effects of other drugs in osteoarthritis

Intra-articular injection of glucocorticoids is a common clinical treatment for relieving symptoms of OA. However, long-term use of glucocorticoids may induce cartilage degeneration and inhibit cartilage growth and ECM synthesis. It was found that melatonin may play a preventive effect on dexamethasone-induced chondrocyte injury through the SIRT1-related signaling pathway (Yang et al., 2017). Researchers have constructed an injectable hydrogel/microparticle system composed of melatonin and methylprednisolone and verified its ability to promote cartilage formation *in vitro* and *in vivo* (Naghizadeh et al., 2021).

## The role of ncRNAs in osteoarthritis and its interaction with melatonin

The regulatory effect of melatonin on OA has been described above. The regulatory effect of melatonin on OA is partly achieved by acting on ncRNAs. At the same time, some ncRNAs are not regulated by melatonin, but they can function on a similar pathway to melatonin. These types of ncRNAs are highlighted below. The related ncRNAs and their expression and regulatory mechanisms for OA are shown in Tables 1, 2, 3.

**TABLE 1:** Summary of differentially expressed miRNAs concerning the regulatory effect of melatonin on OA discussed in this review.

miRNAs	OA influence	MT influence	Target gene	Main influence of miRNAs	Model	References
miR-140	↓	↑	ADAMTS 5	↓ ECM degradation	hBMSCs	Karlsen et al. (2016)
			MMP 13	↓ ECM degradation	Human osteoarthritic chondrocytes	Liang et al. (2020)
			Notch	↑ Chondrocyte differentiation	Cartilage progenitor cells	Si et al. (2019)
			IL1B, IL6, SDC4	↓ Inflammatory mediators and response	hBMSCs	Karlsen et al. (2016)
miR-146a	↑	↓	NRF2	↑ Oxidative stress and apoptosis	Human osteoarthritic chondrocytes	Zhou et al. (2022)
			Tgif1, Camk2d&Ppp3r2	↓ ECM synthesis	Mouse chondrocytes	Zhang et al.(2017)
			TRAF6	↑ Oxidative stress and apoptosis	Human osteoarthritic chondrocytes	Shao et al. (2020)
	↓		Notch	↓ Inflammatory mediators and response	Mouse chondrocytes	Guan et al. (2018)
miR-526b-3p	↓	↑	Smad7	↑ Chondrocyte differentiation	hBMSCs	Wu et al. (2019)
miR-590-5p	↑	↑	Smad7	↑ Chondrocyte differentiation	hBMSCs	Wu et al. (2018)
			FGF18	↑ Oxidative stress and apoptosis	Human osteoarthritic chondrocytes	Jiang et al. (2021b)
miR-122		uncertain	SIRT1	↑ ECM degradation	SW1353	Bai et al. (2020)

Note: Table 1 show the differentially expressed miRNAs concerning the regulatory effect of melatonin on OA discussed in this review. The up and down arrow represent upregulation and downregulation respectively. “OA influence” means changes of miRNA in OA. “MT influence” means the effect of MT on miRNA. This table is based on the findings from various studies cites appropriately in the text. miRNAs, microRNAs; OA, osteoarthritis; MT, melatonin; ADAMTS5, a disintegrin metalloproteinase with thrombospondin motifs 5; ECM, extracellular matrix; hBMSCs, human bone marrow mesenchymal stem cells; MMP 13, matrix metalloproteinase 13; NRF2, nuclear factor-erythroid 2-related factor 2; SIRT1, silent information regulator 1.

**TABLE 2:** Summary of differentially expressed lncRNAs concerning the regulatory effect of melatonin on OA discussed in this review.

lncRNAs	OA influence	MT influence	Target gene	Main influence on OA	Model	References
lncRNA MALAT1	↑	↓	miR-150-5p/AKT	↓ ECM degradation	Human osteoarthritic chondrocytes	Zhang et al. (2018)
				↓ Oxidative stress and apoptosis		
			miR-146a-PI3K/Akt/mTO	↓ Oxidative stress and apoptosis	Human osteoarthritic chondrocytes	Li et al. (2017)
			JNK	↓ ECM degradation	Rat chondrocytes	Gao et al. (2018)
				↓ Oxidative stress and apoptosis	Rat chondrocytes	Gao et al. (2019)
			miR-145/ADAMTS5	↑ ECM degradation	Human osteoarthritic chondrocytes	Liu et al. (2014)
lncRNA MEG3	↓	Uncertain	miR-93/TGFBR2	↓ ECM degradation	Rat chondrocytes	Chen et al. (2021a)
			mir-16/smad7	↑ Oxidative stress and apoptosis	Rat chondrocytes	Xu et al. (2022)
			miR-9-5p/KLF4	↓ Oxidative stress and apoptosis	CHON-001 and ATDC5	Huang et al. (2021)
			P2X3	↓ Inflammatory mediators and response	SW1353	Li et al. (2020)
			TRIB2	↑ Chondrocyte differentiation	SMSCs	You et al. (2019)
lncRNA H19	↓	↑	miR-483–5p/dusp 5	↓ ECM degradation	Rat chondrocytes	Wang et al. (2021a)
				↑ ECM synthesis		
			miR-106b-5p/TIMP2	↓ ECM degradation	Rat chondrocytes	Tan et al. (2020)
			miR-106a-5p	↑ Oxidative stress and apoptosis	Human articular chondrocytes	Zhang et al. (2016)

Note: Table 2 show the differentially expressed lncRNAs concerning the regulatory effect of melatonin on OA discussed in this review. The up and down arrow represent upregulation and downregulation respectively. “OA influence” means changes of lncRNAs in OA. “MT influence” means the effect of MT on lnRNA. This table is based on the findings from various studies cites appropriately in the text. lncRNAs, long non-coding RNAs; OA, osteoarthritis; MT, melatonin; ECM, extracellular matrix; ADAMTS5, a disintegrin metalloproteinase with thrombospondin motifs 5; SMSCs, synovial mesenchymal stem cells.

**TABLE 3:** Summary of differentially expressed circRNAs concerning the regulatory effect of melatonin on OA discussed in this review.

circRNAs	OA influence	MT influence	Target gene	Main influence on OA	Model	References
circRNA 3503	↑	↓	miR-181c-3p	↓ ECM degradation	Human articular chondrocytes	Tao et al. (2021)
			hsa-let-7b-3p	↑ECM synthesis	Human articular chondrocytes	Tao et al. (2021)
circRNA 0045714	↑	Uncertain	miR-218-5p/HRAS	↓ ECM degradation	Human articular chondrocytes	Jiang et al. (2021b)
				↓ Inflammatory mediators and response		
				↑ Oxidative stress and apoptosis		
			miR-193b/IGF1R	↑ ECM synthesis	Human articular chondrocytes	Li et al. (2020)
				↓ Oxidative stress and apoptosis		
			miR-331-3p/PIK3R3	↑ ECM synthesis	Human articular chondrocytes	Ding et al. (2021)
				↓ Inflammatory mediators and response		
				↓ Oxidative stress and apoptosis		

Note: Table 3 show the differentially expressed circRNAs concerning the regulatory effect of melatonin on OA discussed in this review. The up and down arrow represent upregulation and downregulation respectively. “OA influence” means changes of circRNAs in OA. “MT influence” means the effect of MT on circRNAs. This table is based on the findings from various studies cites appropriately in the text. circRNAs, circular RNAs; OA, osteoarthritis; MT, melatonin; ECM, extracellular matrix; IGF1R, insulin-like growth factor 1 receptor.

### MiR-140

MiR-140 is expressed specifically in articular cartilage and plays an important role in cartilage protection. A previous study found that various miRNAs were differentially expressed in Melatonin-treated chondrocytes, so they chose miR-140 as the target for further study and found that melatonin could prevent OA-induced cartilage destruction by promoting the expression of miR-140 and regulating the expression of matrix-degrading enzymes at the post-transcriptional level (Zhang et al., 2019c). Meanwhile, inhibition of miR 140 could counteract the anti-inflammatory effect of melatonin in chondrocytes. IL-1β stimulation inhibited the expression of miR-140 (Zhang et al., 2019c). MiR-140 is widely involved in the regulation of chondrocyte function and can directly downregulate enzymes related to chondrocyte matrix degradation like ADAMTS-5 (Karlsen et al., 2016) and MMP-13 (Liang et al., 2016) at the mRNA level. Furthermore, the expression of miR-140 was increased in chondrogenic hBMSCs (Miyaki et al., 2009). MiR-140 is a strong inducer of the Chondrogenic differentiation of BMSCs (Mahboudi et al., 2018). In fact, miR-140 may activate the cartilage progenitor cells (CPCs) by inhibiting the Notch signaling pathway and promoting the potential of OA cartilage damage repair (Si et al., 2019). TGFBR1, the BMP pathway receptor, is a direct target of miR-140, and up-regulation of miR-140 can inhibit the expression of TGFBR1 and its downstream SMAD1, suggesting that miR-140 can regulate chondrocyte homeostasis through the TGF-β/BMP pathway (Tardif et al., 2013; Zhang et al., 2019c). In addition, miR-140 can inhibit the NF-κB pathway by directly degrading IL1B, IL6, and SDC4 mRNA and binding and inhibiting several distinct points of attack of NF-κB downstream signaling IKBA (Karlsen et al., 2016)**.**

### MiR-146a

It is found that miR-146a knockout aggravates joint degeneration in a mouse model of OA characterized by cartilage degeneration, synovitis, and osteophyte (Guan et al., 2018). Advanced research showed that miR-146a suppressed inflammatory responses in joints by inhibiting Notch signaling. However, miR-146a was significantly upregulated in articular cartilage tissue and serum of OA patients (Skrzypa et al., 2019) and negative regulation in the maintenance of cartilage homeostasis, such as inhibiting cartilage ECM synthesis and promoting chondrocyte apoptosis (Zhang et al., 2017; Shao et al., 2020). Numerous studies have proved the antioxidant effect of melatonin. Zhou et al. (2022) found a novel antioxidative pathway of melatonin in OA chondrocytes: melatonin can upregulate nuclear factor-erythroid 2-related factor 2 (NRF2) protein level by releasing the binding of miR-146a to key antioxidant transcription factor: NRF2 mRNA in early OA cartilage, further enhanced the activity of heme oxygenase 1 (HO-1), an antioxidant enzyme in chondrocytes by the NRF2/HO-1 axis, playing an antioxidant role in chondrocytes (Zhou et al., 2022). However, the regulation mechanism of melatonin on chondrocyte homeostasis through miR-146a still needs to be further studied.

### MiR-526b-3p and miR-590-5p

It was found that melatonin can upregulate miR-526b-3p and miR-590-5p, which can target and inhibit SMAD7, thereby enhancing the phosphorylation of SMAD1 and finally activating the BMP/SMAD pathway to promote the chondrogenic differentiation of hBMSCs (Wu et al., 2018). Melatonin concentration-dependent and reversible by BMP/SMAD pathway inhibitors. The promoting effect of melatonin on cartilage differentiation is concentration-dependent and can be reversed by BMP/SMAD pathway inhibitors. Various ncRNAs can regulate cartilage differentiation through the BMP/SMAD pathway, but their interaction with melatonin has not yet been demonstrated (Lin et al., 2009). In addition, miR-590-5p was found to promote OA progression by targeting fibroblast growth factor18 (FGF18) (Jiang et al., 2021b).

### MiR-122

Melatonin exerted cytoprotective and anti-inflammatory effects in hydrogen peroxide H_2_O_2_)-stimulated human chondrocyte cell line (CHON-001) and rabbit OA model through the SIRT1 pathway (Lim et al., 2012). It was found that overexpression of miR-122 increased the expression of ECM catabolic factors, such as disintegrins, MMPs, and ADAMTS, and inhibited anabolic genes such as collagen II and aggrecan glycan expression. Inhibition of miR-122 expression had the opposite effect. Furthermore, it was identified as a direct target of miR-122, suggesting the potential of the miR-122/SIRT1 axis in regulating the degradation of ECM in OA (Bai et al., 2020). In addition, rhythm genes are also involved in the regulation of the SIRT1 pathway (Yang et al., 2016). Transfection of siRNA targeting SIRT1 resulted in not only a reduction in protein expression of *bmal1* but also a modest increase in *per2* and Rev-Erbα, the key rhythm gene involved in cartilage homeostasis mediated by SIRT1 concomitantly. All miR-122, melatonin and the rhythm gene *bmal1* can protect cartilage through the SIRT1 pathway, suggesting that the three may interact to maintain cartilage homeostasis.

### LncRNA metastasis-associated lung adenocarcinoma transcript 1

Highly sequence-conserved across species and widely studies show that lncRNA metastasis-associated lung adenocarcinoma transcript 1 (MALAT1) has been documented to play a critical role in multiple diseases (Kim et al., 2018; Chen et al., 2021b). Lnc-MALAT1 was upregulated in OA chondrocytes and positively correlated with the severity of OA, and further experiments found that lnc-MALAT1 could target and inhibit miR-150-5p, thereby regulating the proliferation, apoptosis, and ECM degradation of human OA chondrocyte model through the miR-150-5p/AKT3 axis (Zhang et al., 2019d). Similarly, lnc-MALAT1 inhibits miR-146a by targeting and producing similar effects through the miR-146a-PI3K/Akt/mTOR axis (Li et al., 2020). Besides miRNAs, lnc-MALAT1 can also inhibit chondrocyte apoptosis and reduce cartilage ECM degradation by inhibiting the JNK signaling pathway (Gao et al., 2019). Liu et al. (2019) found that IL-1β-stimulated intracellular lnc-MALAT1 in human chondrocytes could target miR-145 and inhibit its function, thereby enhancing ADAMTS5 expression and ultimately leading to enhanced degradation of the chondrocyte ECM (Liu et al., 2019). Lnc-MALAT1 is also an important signaling molecule for regulating drugs on OA (Zhang et al., 2020). Meanwhile, melatonin significantly downregulates lncRNA-MALAT levels in cardiomyocytes (Che et al., 2020). Further research is needed to verify the interaction between melatonin and lncRNA-MALAT in chondrocytes.

### LncRNA maternally expressed 3

Numerous experiments have demonstrated the regulatory role of maternally expressed 3 (MEG3) in OA. And this regulation is mainly achieved by functioning as the ceRNAs of miRNAs. It was found that MEG3 was significantly downregulated in chondrocytes treated with IL-1β. Further research found that MEG3 regulates the miR-93/TGFBR2 axis by targeting miR-93, thereby activating the TGF-β signaling pathway, Regulating IL-1β-induced chondrocyte ECM degradation. A previous study (Chen et al., 2021a) found that MEG3 was significantly downregulated in IL-1β-treated chondrocytes. Further studies found that MEG3 regulates the miR-93/TGFBR2 axis by targeting miR-93, activating the TGF-β signaling pathway, and regulating IL-1β-induced chondrocyte ECM degradation. A previous study found that MEG3 exerted anti-proliferative and pro-apoptotic effects on OA chondrocytes by regulating miR-16 and SMAD7 (Xu and Xu, 2017). However, some studies have reached the opposite result. It is found that MEG3 induces KLF4 expression by inhibiting miR-9-5p, thereby promoting chondrocyte proliferation and migration and inhibiting apoptosis and inflammation. The different regulatory effects of MEG3 on chondrocytes may be caused by differences in experimental subjects (Huang et al., 2021). In addition, LncRNA MEG3 can also exert an anti-OA effect by regulating other genes (Li et al., 2018; You et al., 2019). At the same time, the regulatory effect of melatonin on LncRNA MEG3 has been verified in a variety of diseases, such as atherosclerosis (Zhang et al., 2018), stroke (Chen et al., 2020), and febrile convulsion (Wang et al., 2021b). Melatonin also showed negative regulation of LncRNA MEG production. However, in an experimental diabetic retinopathy cell model, melatonin delays diabetic retinopathy (DR) progression by upregulating MEG3 and inhibiting model cell activation and pro-inflammatory cytokine production via the MEG3/miR-204/SIRT1 axis (Tu et al., 2021). Unfortunately, there is still nothing we know about the interaction of melatonin with lncRNA MEG3 in the regulation of OA.

### LncRNA H19

LncRNA H19 is highly expressed in the peripheral blood of OA patients, is closely related to the occurrence and development of OA, and has a diagnostic value for OA (Zhou et al., 2020). It was found that lncRNA-H19 promoted ECM synthesis and inhibited the expression of degradation-related enzymes through the lncRNA H19/miR-483–5p/Dusp 5 axis, and then activated the Erk and p38 pathways in the cartilage degradation induced by intermittent cyclic mechanical stress (ICMS) (Wang et al., 2020). A previous study found that the level of H19 was significantly downregulated in cartilage samples from OA patients and found that lncRNA H19 may promote chondrocyte proliferation and migration by targeting the miR-106b-5p/TIMP2 axis and inhibit ECM degradation in OA cell experiments (Tan et al., 2020). Zhang et al (2019a) found that lncRNA-H19 could regulate the proliferation and apoptosis of OA chondrocytes treated with IL-1β by targeting miR-106a-5p (Zhang et al., 2019a). In addition, the regulatory effect of melatonin on H19 has also been verified in a variety of diseases, such as melatonin promotes the osteogenic differentiation of BMSCs and inhibits adipogenic differentiation by up-regulation of the H19/miR-541-3p/APN axis (Han et al., 2021). Melatonin promotes the expression of lncRNA H19 in delayed brain injury (DBI) after subarachnoid hemorrhage (SAH) (Xu et al., 2022). However, research on melatonin’s regulatory effects on H19 in OA is still lacking.

### CircRNA 3503

It was found that circRNA 3503 was significantly upregulated in melatonin-treated chondrocytes. Meanwhile, the expression of circRNA 3503 was inhibited by TNF-α or IL-1β (Tao et al., 2021). As sleep-related ncRNAs, circRNA3503 maintains cartilage homeostasis by inhibiting apoptosis and upregulating the expression of genes (aggrecan, Col-II, and SOX9) related to ECM synthesis. However, the researchers found that circRNA3503 was not the direct cause of the protective effect on chondrocytes. Through further experiments, it was speculated and verified that circRNA3503 could promote the expression of PPARGC1A (PGC-1α) by acting as a molecular sponge of miR-181c-3p, thereby releasing the effect of IL-1β on the expression of genes related to CASPASE-3 activation and ECM degradation. Indirect regulation of chondrocyte homeostasis by inhibiting and promoting the expression of ECM synthesis-related gene SOX9 by acting as a molecular sponge for let-7b-3p (Tao et al., 2021).

### CircRNA 0045714

The expression of circ 0045714 in knee cartilage tissues and cells of OA patients was lower than that of normal controls, and circ 0045714 could regulate chondrocyte homeostasis by regulating various miRNAs. It is found that the up-regulation of circ 0045714 inhibited TNF-α-induced chondrocyte growth inhibition, inflammation, and ECM degradation by inhibiting the miR-218-5p/HRAS axis (Jiang et al., 2021a). While melatonin could inhibit collagenase-induced OA (Hong et al., 2014), it suggested that melatonin may interact with circ 0045714 when regulating TNF-α. Li et al. (2017)found that circ 0045714 could regulate the synthesis of ECM by inhibiting the transcriptional activity of miR-193b and promoting the expression of miR-193b target gene insulin-like growth factor 1 receptor (IGF1R) (Li et al., 2017). Ding et al. (2021).up found that circ 0045714 plays a protective role in IL-1β-induced chondrocyte injury by targeting and inhibiting miR-331-3p and then upregulating PIK3R3, which is manifested in alleviating chondrocyte apoptosis, inflammatory response and ECM degradation (Ding et al., 2021).

## Prospect of combined application of melatonin and ncRNAS in osteoarthritis treatment

### The regulatory roles of ncRNAs on osteoarthritis

The therapeutic effects of ncRNAs on OA are complex and diverse. One ncRNA can regulate OA from multiple pathways by acting on one or more target genes (Della Bella et al., 2020). Meanwhile, there are also interactions between different ncRNAs. For example, lncRNAs can act on multiple miRNAs simultaneously, and there are also complex interactions between circRNAs and lncRNAs (Sen et al., 2014; Noh et al., 2018). This further expands the regulatory role of ncRNAs on OA. One ncRNA can regulate OA by acting on multiple target genes, but this regulatory effect is not necessarily consistent. Under the premise of excluding experimental error, there are two possible reasons for this situation: one is that ncRNAs play different regulatory roles in chondrocytes in different physiological or pathological states, the other reason is that ncRNAs can both positively and negatively regulate the OA process, for example, one ncRNA inhibits autophagy in chondrocytes while promoting ECM synthesis. Therefore, determining whether one ncRNA can really protect OA requires further experimental verification, which is also conducive to screening the most suitable ncRNAs for OA treatment.

### The therapeutic effects of melatonin on osteoarthritis

A large number of animal experiments have demonstrated the modulating effect of intra-articular melatonin on OA (Zhang et al., 2019c), and serum melatonin concentrations also appear to play a modulating effect on arthritic disease (Hong et al., 2014). Although previous experiments found that intraperitoneal melatonin aggravated the inflammatory response in rats with collagen-induced arthritis (Bang et al., 2012), pinealectomy reduces the level of local focal inflammation in rats (Vriend and Reiter, 2015). The dual effect of serum melatonin concentration and local inflammation of the joint needs further research on whether increasing serum melatonin can produce a therapeutic effect similar to local injection of melatonin in OA and whether increasing serum melatonin concentrations enhances or diminishes the therapeutic effect of topical melatonin injections. These issues need further research and verification, especially considering that the regulatory effect of melatonin on chronic inflammatory pain, which is one of the most common clinical manifestations of OA patients, has been verified in human trials (Vidor et al., 2013).

Melatonin has been a research hotspot for the past two decades, and its therapeutic potential in various diseases has been found. Recent studies have been focusing on the regenerative potential of melatonin (Majidinia et al., 2018). Melatonin, a pleiotropic hormone with few side effects and easy access, has multiple regulatory effects on cartilage and has potential therapeutic effects on OA-related clinical manifestations and complications such as pain, insomnia, and depression, suggesting that melatonin is one of the best candidates for OA treatment. However, there are also limitations. The antiarthritic effect of melatonin is determined by several factors, including dose, duration of therapy, combined exercise, and duration of the initial intervention (Hong et al., 2017), which means that the dose and duration of melatonin application should be based on the patient’s condition. Precise regulation and regulation of ncRNAs provide a feasible research idea for alleviating the adverse effects of melatonin.

### Combined application of melatonin and ncRNAs in osteoarthritis treatment

Research on the interaction of melatonin and ncRNAs have been carried out in the treatment of other diseases with the corresponding results (Alamdari et al., 2021). However, the application of melatonin in clinical practice can only be established since the corresponding ncRNAs and their signaling pathways are fully exploited. Unfortunately, the existing research is not comprehensive. Studies on the relationship between melatonin and ncRNAs in the treatment and pathogenesis of OA mainly focus on miRNAs, causing studies on other ncRNAs, especially lncRNAs, are still lacking. Although we can infer that lncRNAs also interacts with melatonin in OA from the evidence that lncRNAs and melatonin can regulate the same miRNAs and the interaction between lncRNAs and melatonin in other diseases, further experiments are needed to verify this our hypothesis. The complex relationship between melatonin and ncRNAs has created a new perspective for a better understanding of OA pathogenesis contributing to find new treatment strategies based on endogenous compounds. By modulating certain local ncRNAs levels, we can effectively improve the limitations of melatonin in the treatment of OA and enhanced the power of melatonin achieve better therapeutic efficacy.

## Conclusion

The ncRNAs and melatonin interactome are now considered significant regulators for most aspects of the mechanism and treatment of OA. This review summarizes the interplay between melatonin and several types of regulatory ncRNAs, including miRNAs, lncRNAs, and circRNAs in OA. miRNAs play a regulatory role by directly targeting and inhibiting genes involved in multiple parts of the pathogenesis of OA, while lncRNAs and circRNAs can play an indirect regulatory role through miRNAs in addition to acting on target genes. The interaction between ncRNAs and the one-to-many, many-to-one relationship between ncRNAs and target genes constitute a complex network of OA regulation, and we found that not only does ncRNAs are one important way for melatonin to regulate OA, but also combing ncRNAs or their regulators with melatonin may be a new approach for the treatment of OA in the future. To the best of our knowledge, this is the first review to explore the therapeutic effect of melatonin on OA from the perspective of ncRNAs, however, further researches are required to screen the most suitable ncRNAs for OA treatment and reduce the side effect of long-term melatonin supplementation.

## Glossary

AANAT: arylalkylamine N-acetyltransferase
ADAMTS: a disintegrin metalloproteinase with thrombospondin motifs
ADSCs: adipose tissue-derived stem cells
BMSCs: bone marrow mesenchymal stem cells
ceRNAs: competing endogenous RNAs
CHON-001: human chondrocyte cell line
circRNAs: circular RNAs
CPCs: cartilage progenitor cells
DBI: delayed brain injury
D-gal: D-galactose
DR: diabetic retinopathy
ECM: extracellular matrix
FGF18: fibroblast growth factor18
hBMSCs: human bone marrow mesenchymal stem cells
hMSCs: human mesenchymal stem cells
HO-1: heme oxygenase 1
ICMS: intermittent cyclic mechanical stress
IGF1R: insulin-like growth factor 1 receptor
IL-6: interleukin 6
IVDD: intervertebral disc degeneration
lncRNAs: long non-coding RNAs
MALAT1: metastasis-associated lung adenocarcinoma transcript 1
MEG3: maternally expressed gene3
miRNAs: microRNAs
MMP: matrix metalloproteinase
MSC: mesenchymal stem cells
ncRNAs: non-coding RNAs
NF-κB: nuclear factor kappa-B
NLRP3: nucleotide-binding domain and leucine-rich repeat containing PYD-3
NR1D1: nuclear receptor subfamily 1 group D member 1
NRF2: nuclear factor-erythroid 2-related factor 2
OA: osteoarthritis
PCM: pericellular matrix
PGC-1α: PPARGC1A
p-SMAD 2/3: phosphorylated SMAD 2/3
RANKL: Receptor Activator of Nuclear Factor-κ B Ligand
RNS: reactive nitrogen species
ROS: Reactive oxygen species
SAH: subarachnoid hemorrhage
SIRT1: silent information regulator 1
TGF-β1: transforming growth factor-β
TNF-α: tumor necrosis factor-α
WT: wild-type
